# Fusion Genes and RNAs in Cancer Development

**DOI:** 10.3390/ncrna7010010

**Published:** 2021-02-04

**Authors:** Kenzui Taniue, Nobuyoshi Akimitsu

**Affiliations:** 1 Isotope Science Center, The University of Tokyo, 2-11-16, Yayoi, Bunkyo-ku, Tokyo 113-0032, Japan; 2 Cancer Genomics and Precision Medicine, Division of Gastroenterology and Hematology/Oncology, Department of Medicine, Asahikawa Medical University, 2-1 Midorigaoka Higashi, Asahikawa, Hokkaido 078-8510, Japan

**Keywords:** fusion RNAs, gene fusions, chromosomal rearrangements, trans-splicing, cis-splicing, FISH, RT-PCR, non-RT-based assay, RNA sequence

## Abstract

Fusion RNAs are a hallmark of some cancers. They result either from chromosomal rearrangements or from splicing mechanisms that are non-chromosomal rearrangements. Chromosomal rearrangements that result in gene fusions are particularly prevalent in sarcomas and hematopoietic malignancies; they are also common in solid tumors. The splicing process can also give rise to more complex RNA patterns in cells. Gene fusions frequently affect tyrosine kinases, chromatin regulators, or transcription factors, and can cause constitutive activation, enhancement of downstream signaling, and tumor development, as major drivers of oncogenesis. In addition, some fusion RNAs have been shown to function as noncoding RNAs and to affect cancer progression. Fusion genes and RNAs will therefore become increasingly important as diagnostic and therapeutic targets for cancer development. Here, we discuss the function, biogenesis, detection, clinical relevance, and therapeutic implications of oncogenic fusion genes and RNAs in cancer development. Further understanding the molecular mechanisms that regulate how fusion RNAs form in cancers is critical to the development of therapeutic strategies against tumorigenesis.

## 1. Introduction

Cancer is a disease of the genome [1,2]. Gene fusions or chromosomal rearrangements are an important class of somatic alterations in cancer and can have important roles in the initial steps of tumorigenesis. [3,4,5]. The first cancer-associated chromosomal rearrangement was identified in 1960 as a translocation of chromosomes 9 and 22 [4,6,7]. The abnormally small resulting chromosome, named the Philadelphia chromosome, was found in over 95% of patients with chronic myelogenous leukemia (CML) and consisted of the breakpoint cluster region (*BCR*) gene fused to the second exon of the Abelson murine leukemia viral oncogene homolog 1 (*ABL1*) gene [8,9]. Additional examples of cancer-associated chromosomal aberrations have been identified in other hematological malignancies and sarcomas; for example, mixed lineage leukemia (MLL) fusions, *RUNX1–RUNX1T1* and *PML*–*RARα*, *EWSR1*–*FLI1* and *EVT6*–*NTRK3* [10,11]. Although originally discovered in hematological malignancies, gene fusions are now known to occur in several solid tumor types [7,12,13]. The first fusion gene found in a solid tumor was *CTNNB1*–*PLAG1* in salivary gland adenoma, which is usually benign [12,14,15]. Other fusion genes were soon discovered in solid tumors and other malignancies, including glioblastoma, melanoma, and prostate, breast, ovarian, lung, colorectal, and head and neck cancers [13].

Most of the nonprotein-coding region of the human genome was previously considered to be “junk DNA” [16]. With the advent of massive parallel sequencing technology, these regions in the human genome have been clearly shown to transcribe dynamically and differentially into noncoding RNAs (ncRNAs), such as microRNAs (miRNAs), small nucleolar RNAs (snoRNAs), long ncRNAs (lncRNAs), and circular RNAs (circRNAs) [17,18,19,20]. Accumulating evidence indicates that lncRNAs play critical roles in diverse biological processes, including differentiation, stem cell pluripotency, embryogenesis, pathogenic infection, neurogenesis, proliferation, and tumorigenesis [16,20,21,22,23,24,25,26]. LncRNAs also function in chromatin and genomic structural remodeling, RNA trafficking, RNA stabilization, transcriptional regulation, translation, signal pathway, and protein degradation [27,28,29,30,31]. Expression of lncRNA, miRNA, and snoRNA have shown close correlations with specific chromosomal rearrangements in cancers [10,32,33,34]. Moreover, fusion circRNAs (f-circRNAs) that are generated by chromosome rearrangement contribute to oncogenic roles [35]. Furthermore, the fusion RNA *SLC45A3*–*ELK4*, which regulates cancer cell proliferation, functions as a lncRNA [36]. However, details of mechanisms of the oncogenic roles of these fusion RNAs are unclear.

The ultimate goal of precision medicine in cancer treatment is the development of therapeutic strategies that specifically target cancer cells without affecting normal cells [37]. Targeting oncogenic fusion genes and RNAs specific to cancer tissue for treatment and diagnosis could bring us closer to the approach. Moreover, these fusions are often present at clonal levels within tumors; their generation is frequently the founding genetic abnormality that drives the cancer [37,38]. In this review, we present the function and biogenesis of these fusions and current knowledge regarding their roles in cancer development. We also describe an overview of methodologies for identifying fusion genes and RNAs in cancer development and tumorigenesis. Development of therapeutic strategies that target fusion genes and RNAs, and the study of their mechanisms of production and actions, may provide robust opportunities to eradicate cancers that harbor aberrant genes and RNAs.

## 2. Biological Functions of Fusion Genes and RNAs

Solid tumors and hematopoietic malignancies often have highly complex, unstable genomes. Many gene fusions are random events caused by genetic instability or abnormal splicing machinery [39,40]. These changes at the gene or RNA level are unlikely to result in functional nucleic acids or proteins, as they may occur in regions where there are no known genes [12]. The functions of fusion genes and RNAs are diverse and dependent on the location of the fusion junction. However, the presence of a genomic fusion in a tumor does not necessarily mean that the fusion affects cancer development or tumorigenesis. Fusion RNAs in which the fusion junction is within the protein-coding region may be largely out-of-frame, and such out-of-frame fusions are unlikely to be functional. However, out-of-frame fusion RNAs may function as long noncoding RNAs and may exhibit regulatory functions [41].

An RNA fused between two genes in-frame is translated into a novel fusion protein that may act as a potent oncogenic driver. Kinase genes are often partners in such fusions [7,12]. Kinase fusions often retain kinase activity and result in ligand-independent constitutive activation and enhanced downstream signaling that leads to carcinogenesis [7,12]. Tyrosine kinase fusions that contain kinase-encoding genes, such as *ALK*, *ROS1*, *RET*, *FGFR1/2/3* and *NTRK1*, have been detected in various types of cancer, including glioblastoma, melanoma, and carcinomas of head and neck, breast, lung, prostate, bladder, and thyroid gland [12,40,42,43]. Serine–threonine kinase fusions have also been reported [12,40,43]. These kinase fusions frequently cause activation of signaling pathways that play important roles in cell growth, survival, proliferation, and apoptosis [12,40,42,43]. In addition, kinases are ideal targets for cancer therapy; several inhibitors against kinases, such as ALK and BRAF, have been used to treat cancers with fused genes [12,44].

Fusion of transcription factors usually produces a fusion protein that leads to constitutive activation or an altered target gene, providing aberrant transcriptional machinery and cell transformation [12]. For example, the TMPRSS2–ERG fusion protein, the most common fusion in prostate cancers, mediates overexpression of E26 transformation-specific (ETS) family transcription factors in response to androgen and thus aberrantly activates downstream oncogenes that play important roles in many biological processes, including cell proliferation, angiogenesis, and invasiveness [45,46]. Moreover, the EWSR1–FLI1 fusion protein can gain the ability to bind to the genome and change the transcriptional mechanism [47,48]. Transcription factor fusions can induce a wide range of phenotypic changes that initiate or promote tumorigenesis. However, they have been generally more difficult to work with as therapeutic targets than kinase fusions [37].

## 3. Biosynthesis Patterns of Fusion Genes and RNAs

Fusion RNAs are known to be generated by three mechanisms [7,49,50]. The best-understood of these is chromosomal rearrangement. Two other mechanisms are grouped together as “splicing”. One is trans-splicing, in which exons from two separate RNA transcripts are spliced together. The other is cis-splicing, which involves adjacent genes on the same strand.

RNA splicing is a form of RNA processing in which a newly made precursor messenger RNA (pre-mRNA) is transformed into a mature messenger RNA (mRNA) [51,52]. It has important functions in regulating the RNA and protein diversity observed in organisms [51,52]. Pre-mRNA splicing involves recognizing and removing noncoding regions (intron excision) and the concomitant joining of coding regions (exon ligation) to produce mature mRNA. For many eukaryotic introns, splicing is performed in a series of reactions that are catalyzed by the spliceosome, a complex of small nuclear ribonucleoproteins that incorporates stepwise assembly and disassembly by several hundred proteins and five small nuclear RNAs [53,54,55]. Mechanisms for trans- and cis-splicing between neighboring genes are not well understood. In this section, we review the mechanism of fusion genes and RNA generation that are currently known.

### 3.1. Chromosomal Rearrangement

**Figure 1:**
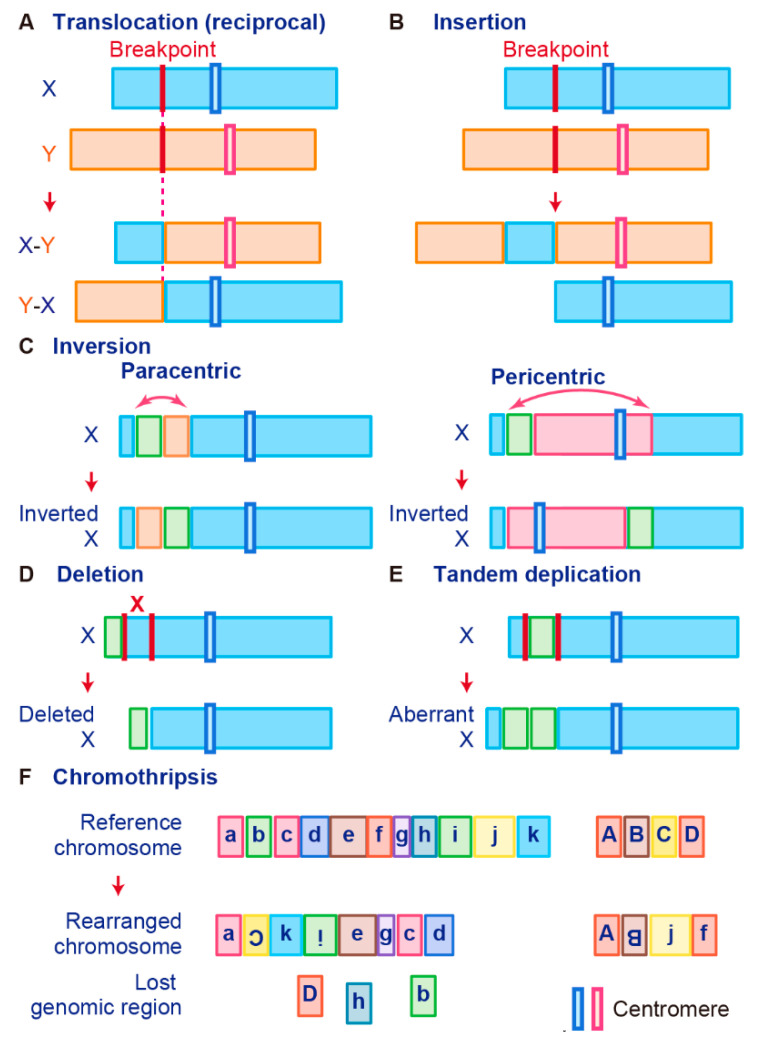
Schematic representation of fusion gene formation by structural chromosome rearrangements. (**A**) Translocation. (**B**) Insertion. (**C**) Inversion. (**D**) Deletion. (**E**) Tandem duplication. (**F**) Chromothripsis.

Gene fusions are usually caused by alterations in genomic structure resulting from DNA damage and by subsequent erroneous recombination and replication [7]. Genomic rearrangements can occur between one or two independent genes through six different known mechanisms: translocation, insertion, inversion, tandem duplication, deletion, and chromothripsis (Figure 1) [7,50,56]. Inversions, tandem duplications, and chromosomal deletions can occur within one or two adjacent genes, whereas translocations and insertions represent large-scale genomic aberrations that result from interactions between distant regions of the genome (interchromosomal rearrangements) or within the same chromosome (intrachromosomal rearrangements) [7,50,56]. As a result, gene fusions can produce aberrant fusion RNAs and proteins that may activate, reduce, or eliminate their original functions.

A chromosomal translocation is an exchange of parts between two nonhomologous chromosomes, also called a reciprocal translocation. Chromosomal translocation can occur anywhere between any two chromosomes (Figure 1A).

The second type of translocation is an insertion. Insertions are caused by transfer of DNA fragments from one region to another within the same chromosome (intrachromosome) or from one chromosome to another (interchromosome). The latter is also known as a nonreciprocal nonmutual translocation (Figure 1B). The *BCR–ABL1*, the first oncogenic fusion gene ever identified, is formed by a reciprocal chromosomal translocation. *BCR–ABL1*, which is generated from translocation t(9; 22) (q34; q11), is characteristic of CML [9] and also found in acute lymphoid leukemia (ALL) [57] and acute myelogenous leukemia (AML) [58]. The fusion gene *BCR–ABL1* has a constitutive tyrosine kinase activity, which leads to sustained stimulation on proliferation of cancer cells [8]. One chromosomal abnormality discovered after *BCR–ABL1* was a consistent nonhomologous balanced translocation between chromosomes 8 and 21 in leukemia patients [59]. This t(8; 21) translocation is one of the most common genetic defects in AML; it gives rise to the *RUNX1–RUNX1T1* fusion gene (previously called *AML1–ETO*) [60,61,62]. The *RUNX1–RUNX1T1* fusion protein interacts with other proteins to repress transcription and induce leukemogenesis in myeloid progenitor cells [60,63].

Promyelocytic leukemia (*PML*)–retinoic acid receptor alpha (*RARα*) is a fusion RNA found in almost 95% of acute promyelocytic leukemia (APL). It is generated from the t(15;17) reciprocal translocation in APL [64,65,66,67,68]. PML is a key component of PML bodies and many proteins have been associated with PML in cells [69]. The PML–RARα protein disrupts PML bodies and induces the formation of dispersed microspeckles with the loss of transcriptional activation ability [70,71]. In addition to leukemia, fusion events also occur in Ewing’s sarcoma (EWS). Many EWS cases involve t(11; 22) or t(21; 22) translocation that fuses the 5′ end of the Ewing’s sarcoma breakpoint region-1 (*EWSR1*) gene to the 3′ end of the *FLI1* or *ERG* gene, which generates the fusion genes *EWS*–*FLI1* or *EWS*–*ERG*, respectively [47,48,72]. These fusion transcription factors upregulate genes related to the cell cycle, invasion, and proliferation pathways [73,74,75,76]. Interestingly, the prion-like domain of EWS–FLI1, which is necessary for phase transitions, induces recruitment of BRG1/BRM-associated factors (BAF) complexes to *GGAA* microsatellites that are frequently found in oncogenes and activate the transcription of target genes [77].

A chromosome inversion occurs when a chromosome undergoes a break or rearrangement within a single chromosome (Figure 1C). There are two types of inversions: paracentric and pericentric. Paracentric inversions do not involve centromeres and both breaks occur in a single chromosome arm, whereas pericentric inversions include a centromere, with one break in each arm (Figure 1C). Many chromosomal rearrangements identified in radiation-induced tumors are known to be paracentric inversions. The most common is the *RET* fusion in papillary thyroid carcinoma, which is present in up to 80% of radiation-related tumors [78]. As another example, in 2007, *EML4*–*ALK* was identified as a novel fusion oncogenic driver of non-small cell lung cancer (NSCLC) [79]. The fusion of *EML4* with *ALK* is caused by an inversion of chromosome 2 (inv2) (p21:p23), by which the kinase domain of the receptor-type tyrosine kinase ALK is placed under the control of the constitutive promoter of *EML4* [80]. This fusion allows cancer transformation by activating downstream reactions in the ALK signaling pathway [80].

Chromosome deletion, which is the fourth type of rearrangement, is an alteration in which a chromosome fragment is lost during DNA replication (Figure 1D). This chromosome deletion causes the deletion of intergenic regions between two genes that are side by side, and leads to formation of fusion genes by aligning two genes that are transcribed in the same direction. An example is *TMPRSS2–ERG,* generated in prostate cancer via an intron deletion between *TMPRSS2* and *ERG* on chromosome 21q22.2-3 [81,82]. Transmembrane serine protease 2 (TMPRSS2) is a prostate-specific androgen-regulated protein, and ETS-related gene (ERG) belongs to the ETS family of transcription factors, which can be oncogenic [81,82,83]. *TMPRSS2*–*ERG* fusion is reportedly associated with higher tumor stage, increased risk of disease progression, and bone metastasis [46,84].

In tandem duplication, a genomic region is duplicated and fused with a gene from the original region (Figure 1E). *FGFR3–TACC3* in glioblastoma is an example of tandem duplication [85]. A tandem duplication also occurs at 7q34 in pilocytic astrocytoma, resulting in a *KIAA1549–BRAF* fusion gene that exhibits constitutive kinase activity. Moreover, a tandem duplication that leads to *C2orf44*–*ALK* fusion occurs in-frame on chromosome 2 in colorectal cancer, resulting in overexpression of the ALK kinase [86].

Tumor cells are associated with high genomic instability, and fusions can occur as a result of complex processes involving several rare and/or complex genetic rearrangements. Chromothripsis, which is the sixth type of rearrangement, occurs when a single chromosome, chromosome region, or a small number of chromosomes are shattered into many fragments and the fragments reassemble incorrectly (Figure 1F). Chromothripsis can produce a large number of fusion genes in a single event [87]. The most typical examples of this characteristic event are *PVT1–MYC* and *PVT1–NDRG1* fusions in medulloblastoma. Chromothripsis in medulloblastoma leads to recurrent translocations that eventually fuse a lncRNA *PVT1* to *MYC*, resulting in a continuous oncogenic effect via MYC amplification [88].

### 3.2. Trans-Splicing

**Figure 2:**
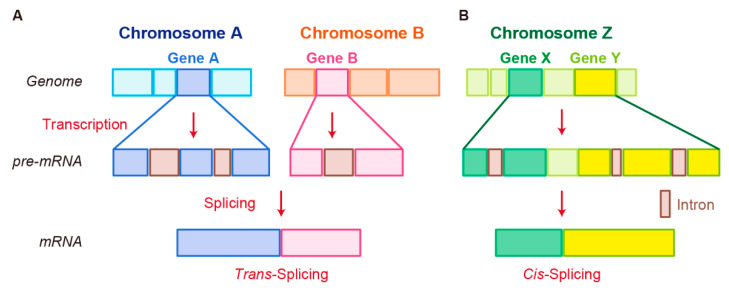
Schematic representation of fusion RNA formation by nonstructural chromosome rearrangements. (**A**) Trans-splicing. (**B**) Cis-splicing.

In trans-splicing, exons from different RNA transcripts are spliced and fused together to produce a mature mRNA (Figure 2A) [89,90,91]. Trans-splicing produces RNAs with exon repetitions or shuffling, as well as RNAs composed of exons transcribed from opposite strands [92,93,94]. The molecular mechanisms of trans-splicing in vertebrates are largely unexplored; however, several models have been proposed [41]. One model is spliceosome-mediated trans-splicing, which uses a canonical splice site of two different primary RNAs. It shows that the spliceosome mechanism, a fundamental component of splicing, can generate trans-spliced fusion RNAs [95,96,97,98].

Examples of trans-spliced fusion RNAs are JAZF1–JJAZ1 (SUZ12) [89] and PAX3–FOXO1 [99]. In both cases, identical fusions were found as structural chromosomal rearrangements from human tumor tissues and RNA trans-splicing from normal human tissue. JAZF1–SUZ12, which is composed of the first 3 exons of JAZF1 and the last 15 exons of SUZ12, canonically resulted from a recurrent translocation t(7;17)(p15;q21) in endometrial stromal tumors. Identical fusion RNA was detected in normal endometrial cells [89]. Whereas PAX3–FOXO1 with t(2;13) translocation was detected in rhabdomyosarcoma, PAX3–FOXO1 fusion RNA produced by trans-splicing was transiently present in cells that underwent differentiation from pluripotent cells into skeletal muscle [99]. In these cases, different mechanisms at genomic or RNA levels may generate identical fusion RNAs, leading to different pathological outcomes.

### 3.3. Cis-Splicing

Another splicing mechanism is cis-splicing, in which two neighboring genes are transcribed into a single precursor RNA by transcriptional read-through, followed by RNA splicing between the exons of the two neighboring genes to complete the fusion (Figure 2B) [7,100]. Although cancer-associated fusion RNAs from cis-splicing are uncommon, some have been reported with clear oncogenic roles. *SLC45A3–ELK4* has been discovered by two independent groups and is a potential biomarker in prostate cancer [101,102]. Several *SLC45A3*–*ELK4* fusions have been reported; the primary form is a fusion of *SLC45A3* exon 1 with the last four exons of *ELK4*. Interestingly, although *SLC45A3–ELK4* functions as a fused lncRNA, its knockdown in cancer cells leads to reduction in cell proliferation, despite its unclear oncogenic roles [36]. *RBM6–RBM5* is found in several types of cancer; its expression is associated with the size of breast tumors [103]. *DUS4L–BCAP29* is found in gastric and prostate cancers and plays a tumor-promoting role in gastric cancer [104,105]. However, *DUS4L–BCAP29* is also present in normal tissues and has a growth-promoting effect in normal as well as cancerous tissues [106].

## 4. Detection of Fusion Genes and RNAs

### 4.1. Guided Approaches to Detect Fusions

The first fusion gene was discovered by chromosome banding techniques in hematologic malignancies. This technique allows each chromosome and chromosome region to be identified on the basis of its unique band pattern, thus subtle rearrangements that were previously undetectable could be found [4,100]. Other techniques that can be used to detect gene fusions include fluorescence in situ hybridization (FISH), polymerase chain reaction (PCR), and comparative genomic hybridization (CGH) array [4,12,100]. FISH can visualize different chromosome structures simultaneously in different colors and greatly improve the depiction of breakpoints in both nondividing cells and on metaphase chromosomes with structural rearrangements [107]. In 1983, PCR technology was developed that would later revolutionize not only biochemistry and molecular biology but diagnostic techniques to detect SARS-CoV-2 [108,109]. However, fusion RNAs that are generated by trans- or cis-splicing cannot be detected by the DNA-based assays described above because they are produced without chromosomal rearrangement. Technologies used to detect these fusion RNAs are RT-PCR, Northern blotting, and RNase protection assays [100].

The cytogenetic approach, which requires indirect and incremental steps, has some important weaknesses [4]. Analysis with chromosome banding techniques requires live cells and rapid transport of tumor samples to the laboratory for cell culture. In addition, highly malignant tumors often have complex genomes, which complicates assigning rearrangements to specific chromosomal bands [4]. The development of array-based platforms for gene expression and copy number profiling in the 1990s provided a new approach to enable the detection of fused genes [110]. Array-based platforms not only enabled a genome-wide view, but provided much higher resolution than chromosome banding techniques, and did not require cell cultures to maintain live cells [110].

### 4.2. Unbiased Approaches to Detect Fusions

The advent of low-cost, high-throughput, massively parallel sequencing technology has revolutionized sequencing by enabling simultaneous generation of thousands to millions of read sequences [111,112,113]. Moreover, sequencing technology makes it possible to identify fusions at the DNA and RNA levels in a single experiment. By simultaneously obtaining detailed and comprehensive information about the genome or transcriptome, identification of structural variants and fusion transcripts without prior information about the cytogenetic characteristics of the tumor tissue is now possible [4]. Detection of chromosomal rearrangements and fusion RNAs has become much easier, compared with the guided approaches described above.

Advances in both sequence technology and computational capabilities have enabled discovery of novel fusion gene or RNA candidates through bioinformatics techniques. However, whereas the sequencing technology is highly sensitive and allows for detection of rare events, it is also error-prone [114,115]. Sequencing errors may be introduced before sequencing or arise from use of a sequencing platform [115]. In addition, several bioinformatics pipelines have been developed to facilitate the identification of chimeric transcripts; each of them has its own features, strengths, and weaknesses [116,117,118]. Algorithms for detecting fusion RNAs differ considerably in their sensitivity and specificity [119], and both false positive and false negative findings from these tools are common [116,117,118]. Therefore, candidate fusion RNAs obtained through these tools should be experimentally validated using guided approaches. Experimental verification of potential fusions is usually performed using RT-PCR reactions, utilizing primers that span the junction positions of the fusion RNAs. However, template switching and creation of false fusions by stem loops can occur during the RT step of cDNA preparation [120,121]. Therefore, fusions detected by sequencing technology should be validated using non-RT-based assays, such as RNase protection assays and Northern blot analysis [122].

### 4.3. Databases for Fusion Genes and RNAs

Sequencing technology to detect cancer-associated fusion genes and RNAs has processed established cell lines, followed by a wide range of primary tissue samples from several cancer types, including carcinomas of the breast, colon, lung, prostate, and uterus, as well as leukemias and lymphomas [101,123,124,125,126,127,128,129]. The Pan-Cancer Analysis of Whole Genomes (PCAWG) Consortium of the International Cancer Genome Consortium (ICGC) and The Cancer Genome Atlas (TCGA) have also detected various fusions in cancer tissue genomes [5,130,131,132]. Moreover, thousands of fusion RNAs and genes are now listed in several databases, including Mitelman, ChimerDB, FusionGDB, FusionGDB, and others (Table 1) [49,133]. However, many of these newly identified candidates have not yet been experimentally validated, and some may represent artifacts of the sequencing processes. Moreover, information on fusion transcripts in these databases lacks uniformity [133].

## 5. Clinical Relevance of Fusion Genes

As gene and RNA fusions tend to be tumor-specific, they can be used as biomarkers to identify cancer types [101]. *BCR–ABL1* has been widely used as a biomarker and prognostic factor in patients with ALL [134], and its specific inhibitor, Glivec (imatinib), has been effectively used to target CML and ALL with *BCR*–*ABL* fusions [135]. *EML4–ALK* is used as a biomarker in NSCLC, which was treated with ALK inhibitors to improve patients’ prognoses [136]. In addition, *TMPRSS2–ERG* gene fusions are expressed in early-phase prostate cancer and allow for prognostic evaluation of patients with prostate cancer [137]. Similarly, as the *EVT6*–*NTRK3* fusion is found in 92% of human secretory breast cancers, it is defined as a diagnostic biomarker [138]. However, although many fusion genes and RNAs have been discovered in cancers and reported as biomarkers, fusions are also present in normal tissues and cells [139,140,141] and may have important functions in normal physiology.

## 6. Conclusions

Targeting the oncogenic fusion proteins that are present only in cancers is a promising strategy for the development of new cancer therapeutics. This promise needs to be realized because many fusion-driven cancers have particularly poor prognoses. Clinical application of therapies that directly target fusion proteins, such as imatinib in CML, has dramatically altered clinical outcomes. Furthermore, the focus of research has expanded to include fused ncRNAs such as *PVT1–MYC* and *SLC45A3–ELK4*. In the future, therapies will likely be established that target specific fusion RNAs, including antisense oligos (ASOs), siRNAs, or microRNAs. These nucleic acid analogues undergo complementary base-pairing with their targets to promote endogenous RNA degradation and inhibit its translation. Altering the chemical composition of the phosphate backbone and sugar components of oligonucleotides has produced greater binding affinity and in vivo stability, leading to improved cellular uptake and release of these nucleic acid analogues. In addition to developing these technologies, elucidating upstream mechanisms that regulate fusion RNA expression and generation is necessary. Therefore, greater understanding of the mechanisms that underly fusion RNA regulation in cancers, with and without chromosomal rearrangements, is critical.

Advances in sequencing technology have accelerated fusion RNA research, and the implementation of large-scale sequencing projects such as TCGA and PGAWG allow identification of rare oncogenic fusions in a variety of cancer types. Furthermore, future advances in sequencing technology will accelerate the pace of chimeric RNA research. The advent of new sequencing technologies, such as low-cost whole-genome sequencing, long-read sequencing technologies [142], and single-cell full-length total RNA sequencing [143], will enable us to discover more fusion RNAs and elucidate their relationship to cancer heterogeneity. Ongoing research in this area can elucidate new mechanisms of cancer development and tumorigenesis, which may contribute to significant improvements in therapeutic and diagnostic techniques for cancer.

## Figures and Tables

**Table 1:** Available databases hosting fusion transcripts.

Database Name	Tumor	Non- Tumor	First Release	Last Update	Current Version	Total Fusions	URL
Mitleman	Yes	No	1983	2020		32,578	https://mitelmandatabase.isb-cgc.org
ChimerDB	Yes	Yes	2006	2020	4.0	67,610	https://www.kobic.re.kr/chimerdb_mirror/
FusionGDB	Yes	No	2019	2020		43,895	https://ccsm.uth.edu/FusionGDB/
COSMIC	Yes	No	2004	2020	92	19,369	https://cancer.sanger.ac.uk/cosmic
ChiTaRs	Yes	Yes	2012	2019	5.0	23,167	http://chitars.md.biu.ac.il/
Tumor Fusion Gene Data Portal	Yes	Yes	2015	2018		20,731	https://www.tumorfusions.org/
FusionHub	Yes	Yes	2018	2018		150,699	https://fusionhub.persistent.co.in
TICdb	Yes	No	2007	2013	3.3	1374	https://genetica.unav.edu/TICdb/
dbCRID	Yes	Yes	2010	2010	0.9	2643	http://c1.accurascience.com/dbCRID/

## Data Availability

Data sharing not applicable.
